# Liraglutide Overdose-Induced Acute Pancreatitis

**DOI:** 10.7759/cureus.21616

**Published:** 2022-01-25

**Authors:** Aljawhara R AlSaadoun, Tariq R AlSaadoun, Abdullah K Al Ghumlas

**Affiliations:** 1 Family and Community Medicine, King Fahad University Hospital/Imam Abdulrahman Bin Faisal University, Dammam, SAU

**Keywords:** medical weight loss, liraglutide, saxenda, glp-1ras, iatrogenic, complications, overdose, pancreatitis, overweight, obesity

## Abstract

Liraglutide, a long-acting cardioprotective glucagon-like peptide (GLP)-1 analog, is effective for medical weight loss and glycemic control in type 2 diabetes. It is generally well tolerated with mild side effects. There are few reports on complications from Liraglutide overdose. The aim of this paper is to report the case of a 25-year-old healthy female who presented with acute pancreatitis secondary to Liraglutide overdose and to review the current literature on Liraglutide used for obesity management. The current literature examining the association between acute pancreatitis and Liraglutide use, and Liraglutide overdose are inconclusive. Further research is recommended.

## Introduction

Long-acting glucagon-like peptide (GLP-1) analogs, such as Liraglutide, are effective for obesity management and glycemic control. GLP-1 analogs enhance pancreatic beta-cell insulin secretion, delay gastric emptying, and enhance satiety. An overdose of such medication may result in generalized gastrointestinal symptoms like nausea, vomiting, hypoglycemia, and pancreatitis. However, Liraglutide overdose and its association with hypoglycemia and pancreatitis need further elucidation. This paper reports a case of Liraglutide overdose-induced acute pancreatitis.

## Case presentation

A 25-year-old single female with no medical or surgical history presented to the emergency room with a one-day history of progressively worsening sharp epigastric abdominal pain with radiation to the back associated with nausea and non-bloody, nonbilious emesis. The pain was exacerbated with lying supine, but worsened with positional changes, and was unrelieved by paracetamol. The review of systems was negative for fevers, jaundice, pruritis, diarrhea, anorexia, insomnia, mood changes, exposure to sick contacts, trauma, or recent travel. She reported regular menses. The family history was notable for gallstones, but negative for diabetes, hypertension, cancer, and cardiac diseases or autoimmune diseases. She denied any allergies to medications, tobacco use, heavy alcohol use, illicit drug use, or sexual activity.

The patient reported a two-month history of unsupervised Liraglutide use for medical weight loss. She had obtained the medication without a prescription and had progressively increased the dose until she reached 3 mg, at which point she could not tolerate it due to nausea and vomiting. The medication was discontinued for a month. She resumed the medication at 2.4 mg on the day of presentation to the emergency room, with the onset of symptoms soon thereafter.

On initial examination, the patient was conscious, alert, and oriented to time, place, and person. She was lying in bed in severe pain. The cardiopulmonary exam was unremarkable. Abdominal examination demonstrated diffuse tenderness with focal intensification in the epigastrium with negative Murphy’s sign. The extremity exam was negative for clubbing, cyanosis, or edema.

As per the patient, she went to a different facility before presenting to our hospital and lipase was more than 900 and amylase was more than 200. However, the patient’s vital signs and laboratory parameters are summarized in Table 1.

**Table 1:** Patient vitals and laboratory parameters in the emergency department. T: Temperature, HR: Heart Rate, RR: Respiratory Rate, BP: Blood Pressure, spO2 RA: Oxygen Saturation on Room Air, BMI: Body Mass Index, CBC: Complete Blood Count, WBC: White Blood Cell Count, HGB: Hemoglobin Test, HCT: Hematocrit Test, PLT: Platelet (Thrombocyte) Count Test, Na: Blood Sodium Level Test, K: Blood Potassium Level Test, CO_2_: Blood Carbon Dioxide Level Test, Cl: Blood Chloride Level Test, BUN: Blood Urea Nitrogen Test, Cr: Creatinine Tests, Ca: Blood Calcium Level Test, T Bili: Total Bilirubin Blood Test, D Bili: Direct Bilirubin Blood Test, Alk Phos: Alkaline Phosphatase Blood Test, LDH: Lactate Dehydrogenase Test, SGOT: Serum Glutamic-Oxaloacetic Transaminase Test, SGPT: Serum Glutamic Pyruvic Transaminase Test, GGTP: Gamma-Glutamyl Transferase Test, Beta HCG: Beta Human Chorionic Gonadotropin Test, HbA1C: Hemoglobin A1c Test.

Vitals								
T 36.9 C	HR 74	RR 20	BP 100/60	SpO2 100% RA	BMI 27		
Labs								
CBC		RFT		LFT		Other	
WBC	12.1	Na	139	T Bili	1.4	Beta HCG	<2.30
HGB	13.4	K	4.2	D Bili	0.4	Lactate	0.88
HCT	43	CO_2_	20	Alk Phos	67	Glucose	77
PLT	550	Cl	105	LDH	238	HbA1c	5.0
		BUN	9	SGOT	22		
		Cr	0.68	SGPT	22		
		Ca	9.9	GGTP	14		
		Anion Gap	14	Lipase	284		
				Amylase	233		

**Figure 1:**
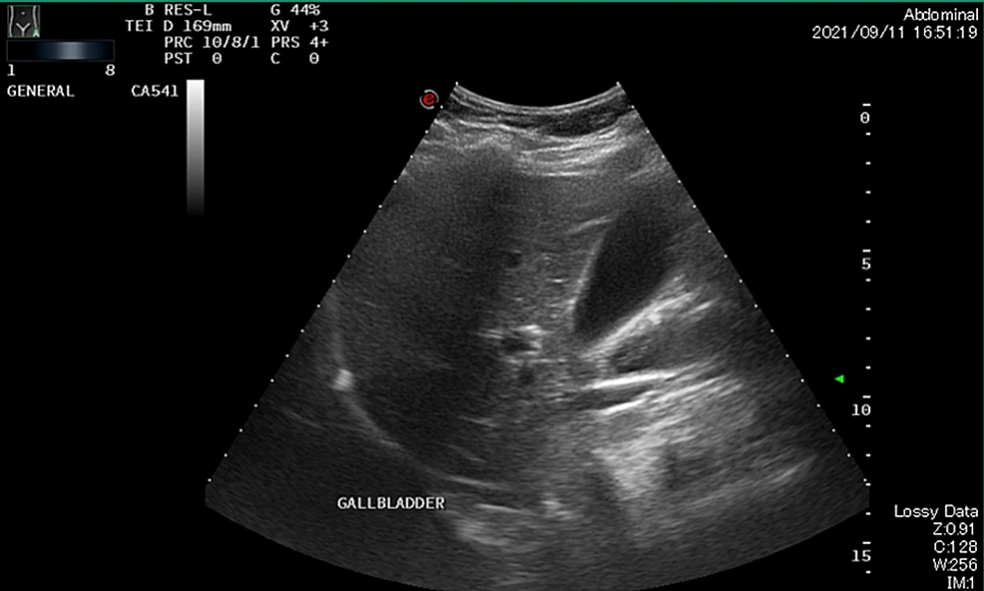
Normal gallbladder with wall thickness measuring 0.3 cm. No cholelithiasis or pericholecystic fluid, and homogenous hepatic parenchyma.

**Figure 2:**
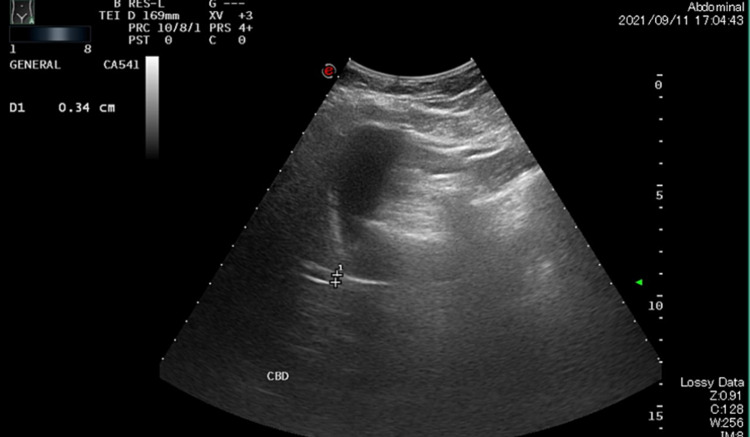
No intrahepatic biliary ductal dilatation. The visualized part of the CBD is normal, with a diameter of 0.3 cm. CBD: Common Bile Duct

Imaging was performed as well. Abdominal and chest radiographs were unremarkable. Abdominal ultrasound was negative for cholelithiasis, cholecystitis, or biliary ductal dilatation (Figures 1, 2). Further, no computed tomography was ordered for the patient.

The patient was admitted to the general surgery services as a case of acute pancreatitis for symptomatic management. She was managed by bowel rest, analgesia, intravenous fluids, antibiotics, and Clexane. The patient showed improvement within 48 hours and was safely discharged with educational materials and follow-up.

## Discussion

GLP-1 receptor agonists (GLP-1 RAs) mediate their effects via receptors expressed in the pancreas, gastrointestinal tract, kidneys, lungs, heart, and brain [1-3]. GLP-1 RAs were authorized for the treatment of diabetes mellites type 2 in 2005. The various GLP-1 RAs have different dosing schedules. Some GLP-1 RAs are injected once daily, such as Lixisenatide (Adlyxin) and Liraglutide (Saxenda), whereas others follow a twice-daily or once-weekly dosing (Semaglutide) [1]. Furthermore, research from 2016 demonstrates that GLP-1 RAs prevent adverse cardiac events such as stroke and myocardial infarction [1].

The class of medications is generally well-tolerated, and the side-effect profile is mostly gastrointestinal in nature (i.e., nausea, vomiting, and diarrhea). Injection site reactions have been reported, and they are more common with Exenatide formulations and Albiglutides. Reports of hypoglycemic episodes are rare [3]. Pancreatitis is a suspected side-effect of GLP-1 RAs; however, few studies and case reports of GLP-1 RA overdose and its association with acute pancreatitis have been described [4]. A recent case report of autopsy demonstrated pathologic pancreatic changes in patients with diabetes who were treated with incretin-based drugs such as Liraglutide [5]. Furthermore, a recent meta-analysis had near-unanimous reporting of adverse gastrointestinal symptoms suggestive of pancreatitis, but there was no formal diagnosis of acute pancreatitis in the acute phase of illness or in the follow-up period [4-16]. Given these considerations, future research, and evaluation of the short- and long-term consequences of GLP-1RA use and overdose are highly recommended [17].

## Conclusions

GLP-1 RA, Liraglutide. is a widely used medication for the treatment of type 2 diabetes mellitus. Also, Liraglutide is effective for weight reduction, glycaemic control, and prevention of adverse cardiac events. Common side effects of Liraglutide are nausea, vomiting, and gastrointestinal discomfort. However, hypoglycemia and pancreatitis are reported but not proven to be associated with Liraglutide overdose. Therefore, further investigation is necessary to determine an association between Liraglutide, Liraglutide overdose, and acute pancreatitis.

## References

[1] Nauck MA, Quast DR, Wefers J, Meier JJ (2021). GLP-1 receptor agonists in the treatment of type 2 diabetes - state-of-the-art. Mol Metab.

[2] Knudsen LB, Lau J (2019). The discovery and development of Liraglutide and semaglutide. Front Endocrinol (Lausanne).

[3] Madsbad S (2016). Review of head-to-head comparisons of glucagon-like peptide-1 receptor agonists. Diabetes Obes Metab.

[4] Nafisah SB, Almatrafi D, Al-Mulhim K (2020). Liraglutide overdose: a case report and an updated review. Turk J Emerg Med.

[5] Chalmer T, Almdal TP, Vilsbøll T, Knop FK (2015). Adverse drug reactions associated with the use of liraglutide in patients with type 2 diabetes--focus on pancreatitis and pancreas cancer. Expert Opin Drug Saf.

[6] Inzucchi SE, Bergenstal RM, Buse JB (2012). Management of hyperglycemia in type 2 diabetes: a patient-centered approach: position statement of the American Diabetes Association (ADA) and the European Association for the Study of Diabetes (EASD). Diabetes Care.

[7] Verspohl EJ (2009). Novel therapeutics for type 2 diabetes: incretin hormone mimetics (glucagon-like peptide-1 receptor agonists) and dipeptidyl peptidase-4 inhibitors. Pharmacol Ther.

[8] Patel DK, Stanford FC (2018). Safety and tolerability of new-generation anti-obesity medications: a narrative review. Postgrad Med.

[9] Bode SF, Egg M, Wallesch C, Hermanns-Clausen M (2013). 10-fold liraglutide overdose over 7 months resulted only in minor side-effects. J Clin Pharmacol.

[10] Nakanishi R, Hirose T, Tamura Y, Fujitani Y, Watada H (2013). Attempted suicide with liraglutide overdose did not induce hypoglycemia. Diabetes Res Clin Pract.

[11] Elmehdawi RR, Elbarsha AM (2014). An accidental liraglutide overdose: case report. Libyan J Med.

[12] Bowler M, Nethercott DR (2014). Two lessons from the empiric management of a combined overdose of liraglutide and amitriptyline. A A Case Rep.

[13] Solverson KJ, Lee H, Doig CJ (2018). Intentional overdose of liraglutide in a non-diabetic patient causing severe hypoglycemia. CJEM.

[14] Krentz AJ, Fujioka K, Hompesch M (2016). Evolution of pharmacological obesity treatments: focus on adverse side-effect profiles. Diabetes Obes Metab.

[15] Drucker DJ, Sherman SI, Bergenstal RM, Buse JB (2011). The safety of incretin-based therapies--review of the scientific evidence. J Clin Endocrinol Metab.

[16] Nyborg NC, Mølck AM, Madsen LW, Bjerre Knudsen L (2012). The human GLP-1 analog liraglutide and the pancreas: evidence for the absence of structural pancreatic changes in three species. Diabetes.

[17] Alves C, Batel-Marques F, Macedo AF (2012). A meta-analysis of serious adverse events reported with exenatide and liraglutide: acute pancreatitis and cancer. Diabetes Res Clin Pract.

